# Clonal Elimination of the Pathogenic Allele as Diagnostic Pitfall in *SAMD9L*-Associated Neuropathy

**DOI:** 10.3390/genes13122356

**Published:** 2022-12-14

**Authors:** K. Eggermann, R. Meyer, M. Begemann, D. Dey, E. Bültmann, I. Kurth, G. C. Korenke, C. Knopp

**Affiliations:** 1 Institute for Human Genetics and Genomic Medicine, Medical Faculty, RWTH Aachen University, 52074 Aachen, Germany; 2 Institute of Diagnostic and Interventional Neuroradiology, Hannover Medical School, 30625 Hannover, Germany; 3 Department of Neuropediatrics, University Children’s Hospital, Klinikum Oldenburg, 26133 Oldenburg, Germany

**Keywords:** *SAMD9L*, CMT, HMSN, copy number neutral loss of heterozygosity, scatter plot, mosaicism

## Abstract

Background: Heterozygous gain-of-function variants in *SAMD9L* are associated with ataxia-pancytopenia syndrome (ATXPC) and monosomy 7 myelodysplasia and leukemia syndrome-1 (M7MLS1). Association with peripheral neuropathy has rarely been described. Methods: Whole-exome sequencing (WES) from DNA extracted from peripheral blood was performed in a 10-year-old female presenting with demyelinating neuropathy, her similarly affected mother and the unaffected maternal grandparents. In addition to evaluation of single nucleotide variants, thorough work-up of copy number and exome-wide variant allele frequency data was performed. Results: Combined analysis of the mother’s and daughter’s duo-exome data and analysis of the mother’s and her parents’ trio-exome data initially failed to detect a disease-associated variant. More detailed analysis revealed a copy number neutral loss of heterozygosity of 7q in the mother and led to reanalysis of the exome data for respective sequence variants. Here, a previously reported likely pathogenic variant in the *SAMD9L* gene on chromosome 7q (NM_152703.5:c.2956C>T; p.(Arg986Cys)) was identified that was not detected with standard filter settings because of a low percentage in blood cells (13%). The variant also showed up in the daughter at 32%, a proportion well below the expected 50%, which in each case can be explained by clonal selection processes in the blood due to this *SAMD9L* variant. Conclusion: The report highlights the specific pitfalls of molecular genetic analysis of *SAMD9L* and, furthermore, shows that gain-of-function variants in this gene can lead to a clinical picture associated with the leading symptom of peripheral neuropathy. Due to clonal hematopoietic selection, displacement of the mutant allele occurred, making diagnosis difficult.

## 1. Introduction

Heterozygous pathogenic variants in *SAMD9L* have been described in a spectrum of multisystem disorders, including non-syndromic (familial) myelodysplastic syndrome with monosomy 7 (OMIM #252270) [1,2,3], *SAMD9L*-mediated autoinflammatory disease [4], Ataxia-pancytopenia syndrome (OMIM #159550) [5,6,7,8,9,10,11], and Spinocerebellar ataxia 49 (OMIM #619806) [12].

Ataxia-pancytopenia syndrome (ATXPC) is characterized by cerebellar ataxia and atrophy, nystagmus, mild pyramidal signs and white matter abnormalities in addition to hematological abnormalities [5,6,7]. Eight families with ATXPC syndrome showing a variable hematological and neurological phenotype have been described so far [5,6,7,8,9,10,11]. However, an association with peripheral neuropathy has rarely been mentioned, and initial clinical presentation of a pathogenic *SAMD9L* variant as demyelinating polyneuropathy has been reported only once [9]. Members of a Spanish multigenerational family affected by SCA49 and a pathogenic *SAMD9L* variant segregating with the disease showed horizontal and vertical gaze-evoked nystagmus and hyperreflexia as initial clinical signs. In the further course of the disease, most affected family members showed neurological abnormalities similar to patients with ATXPC syndrome, early diffuse cerebral demyelination and axonal sensory polyneuropathy but no hematological abnormality [12].

Pathogenic germline variants in ATXPC syndrome and myelodysplastic syndrome with monosomy 7 represent gain-of-function variants as they increase suppression of cell proliferation [3,7,13]. In blood cell lines, this causes pancytopenia and genetic pressure not to express the mutant allele. In carriers of pathogenic *SAMD9L* germline variants, multiple mechanisms of clonal escape have been described [14] and may complicate genetic diagnosis.

Our case report describes a variant in *SAMD9L* as cause of a demyelinating neuropathy and very mild multisystemic involvement and points to the specific pitfalls of next-generation sequencing analysis of *SAMD9L* due to a loss of a pathogenic variant in blood samples.

## 2. Materials and Methods

### 2.1. Case Presentation

**Figure 1:**
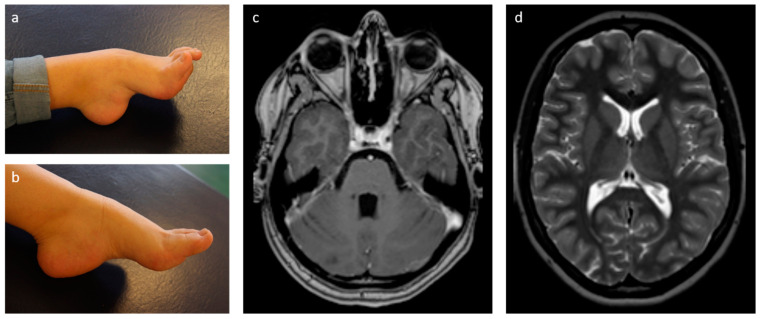
Pes cavus deformity in daughter (**a**) and mother (**b**) with a pathogenic variant in *SAMD9L*. (**c**) Cerebral MRI of the daughter at the age of 15 years showed some minimally accentuated cerebellar fissures on axial T1 weighted images. (**d**) The supratentorial brain was unremarkable according to age as shown on the axial T2 weighted images.

The 10-year-old girl had presented with non-progressive high arched feet since her pre-school years in the neuropediatric department. Developmental milestones were reached on time, and she attended high school with good performance. Measurements for size, weight and head circumference were within the normal range. On examination there was no muscle atrophy, but contractures of Achilles tendons and problems walking on the heels. There were no clinical signs of ataxia or balance problems. Slightly increased muscle tonus in the legs compared to the arms and brisk patellar reflexes were noticed. Oculomotor function was normal. Motor nerve conduction velocity on the right side was reduced (N. medianus 30.4 m/s (ref. 51.2–58 m/s), N. ulnarius 33.3 m/s (ref. 58.0–78.0 m/s), N. peroneus 21.4 m/s (ref. 45.0–74.0 m/s), N. tibialis 20.2 m/s (ref. 48.2–56.6 m/s) with still normal distal motor latencies. Additionally, reduced compound motor nerve action potentials were present predominantly in the legs. MRI of the brain (cMRI) was initially reported normal at the age of 10 and 15 years. However, careful reevaluation of both cMRIs revealed very mild abnormalities of the cerebellum with some mildly accentuated cerebellar fissures without signs of progression. Otherwise, the brain was unremarkable according to age (Figure 1). Spinal MRI was normal. Full blood count showed mild thrombocytopenia (150,000/µL; normal 171,000–388,000/µL) at the age of 14 years. *PMP22* dosage analysis was normal and a next-generation sequencing panel of eleven genes associated with hereditary motor and sensory neuropathy (HMSN, also referred to as Charcot Marie Tooth disease, CMT) did not reveal a causative variant.

The 37-year-old mother was similarly affected by neuropathy. She reported problems in sports since childhood and presented with less-pronounced arched feet compared to her daughter’s. There were normal reflexes, no signs of ataxia and normal oculomotor function. Full blood count was normal, and no history of hematological abnormalities was reported. Nerve conduction studies demonstrated reduced conduction velocities with normal distal motor latencies and reduced compound motor nerve action potentials predominantly in the legs. Her parents were healthy and there were no other affected family members.

### 2.2. Genetic Investigation

**Figure 2:**
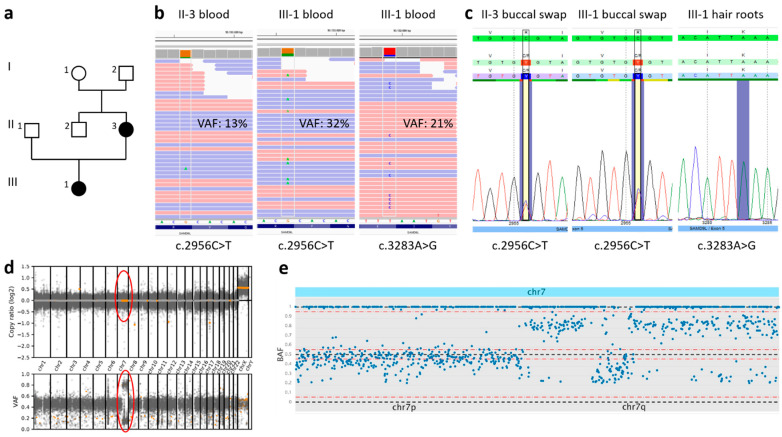
(**a**) Family tree. (**b**) Whole-exome sequencing of DNA extracted from peripheral blood of mother (II-3) and daughter (III-1) showing mosaicism for the variant c.2956C>T; p.(Arg986Cys) with a variant allele frequency (VAF) of 13% (II-3) and 32% (III-1), respectively. The daughter additionally carried a rare somatic missense variant c.3283A>G; p.(Lys1095Glu) with a VAF of 21%. The variant c.2956C>T was not present in the grandparents I-1 and I-2 in leukocyte-derived DNA in exome sequencing data. (**c**) Sanger sequencing of DNA extracted from different tissues of II-3 and III-1 confirmed the germline status of the variant c.2956C>T and somatic status of the variant c.3283A>G. (**d**) Genome wide scatter plot illustration with the detection of UPD7q of the mother (circles). (**e**) A scatter plot (generated by SNPitty) of the b-allele frequency from chr 7 of II-2 showed copy number neutral loss of heterozygosity of 7q caused by stretches of isodisomic uniparental disomy (UPD). UPD7q correlated with a concordant decrease in the variant allele frequency of the *SAMD9L* variant, evident in both WES and Sanger sequencing data.

Whole-exome sequencing was performed analyzing DNA from peripheral blood samples of the mother (II-3), daughter (III-1), and the healthy maternal grandparents (I-1 and I-2) (Figure 2a). Exome enrichment was done using the Lotus™ DNA Library preparation kit (IDT, Coralville, IA, USA) according to the manufacturer’s protocol. Sequencing was performed on a NextSeq500 Sequencer with 2 × 75 cycles on a high-output flow cell (Illumina, San Diego, CA, USA). FastQ-files were generated with bcl2fastq2 (Illumina). An in-house pipeline based on SeqMule was used for alignment and variant calling utilizing GATKLite, SAMtools and FreeBayes [15,16,17,18]. Filtering and annotation of variants was performed using KGGSeq [17]. Average coverage in target region for all samples was between 73 and 124. Copy number variation (CNV) analysis of WES data and illustration by a genome-wide scatter plot of copy number distribution and b-allele frequency was generated by CNVkit (https://cnvkit.readthedocs.io/en/stable/ (accessed on 8 August 2022)). A scatter plot of the b-allele frequency from chr 7 was generated by SNPitty [19]. Sanger sequencing was performed on DNA extracted from different non-blood tissues (hair roots, fingernail, buccal swab) of the mother and daughter and from a peripheral blood sample of the father (II-1).

## 3. Results

Initial analysis of the variants shared by the mother and the daughter did not reveal any obvious causative variants. In contrast, variant evaluation of the girl’s sample in comparison with her healthy grandparents showed a previously reported likely pathogenic variant in the *SAMD9L* gene (NM_152703.5:c.2956C>T; p.(Arg986Cys)) (Figure 2b). Re-analysis of the mother’s data showed low-grade mosaicism for this variant with an allele frequency (VAF) of 13% in peripheral blood (Figure 2b), which was below the threshold for variant calling. Sanger sequencing from DNA extracted from buccal swabs of mother and daughter, fingernails of the mother and hair roots of the daughter showed the presence of the variant in non-blood tissues in both probands (Figure 2c). There was no hint that one of the grandparents is a carrier of the *SAMD9L* variant.

In the daughter’s peripheral blood sample, another *SAMD9L* missense variant (NM_152703.5:c.3283A>G; p.(Lys1095Glu)) that has not yet been reported in literature or in large databases and is predicted to be damaging by several in silico prediction tools (*SIFT*: deleterious (score: 0.04), *PolyPhen2* HDivPred: possibly damaging (score: 0.873), CADDphred: 24.7) was detected with a VAF of 21% (Figure 2b). This variant was not inherited from her parents and was not found in any additional tissues, indicating a somatic origin (Figure 2c).

In the mother, a scatter plot of copy number and b-allele frequency showed a copy number neutral loss of heterozygosity (LOH) of chromosome 7 for most of the long arm, elucidating the finding of somatic mosaicism for the likely pathogenic variant (Figure 2d,e). The read depth of single nucleotide polymorphisms was maintained for the whole chromosome 7 and clarified that LOH was due to a uniparental disomy (UPD) and not a deletion of 7q. Thorough re-evaluation of WES data of the mother did not reveal further variants in *SAMD9L*.

## 4. Discussion

The here identified *SAMD9L* germline variant p.(Arg986Cys) has already been described in association with ATXPC [6,7] as well as familial myelodysplastic syndrome [2,20] and in one isolated case with the clinical diagnosis of hereditary and sensory neuropathy due to demyelinating neuropathy as the initial clinical sign [9].

In our patients, the combination of high arched feet and demyelinating neuropathy in two consecutive generations suggested the presence of HMSN, whereby the additional occurrence of brisk patellar reflexes was an unusual finding and prompted further investigation. Consequently, cMRI was performed, which, however, was only classified as mildly abnormal after reevaluation.

The intra- and interfamilial heterogeneity regarding the neurologic and hematologic manifestations of individuals carrying disease-causing gain-of-function variants in *SAMD9L* is not fully understood, but for the variable hematological outcome an association with a different mechanism of clonal escape from *SAMD9L* germline pathogenic variants has been described before [14,21]. Pathogenic *SAMD9L* variants represent gain-of-function variants and have an adverse effect on cell proliferation which in turn causes pancytopenia and pressure to eliminate the mutant *SAMD9L* allele. Clonal escape from pathogenic *SAMD9L* missense variants including complete or partial loss of chromosome 7, UPD7q and truncating somatic *SAMD9L* variants may occur in carriers of pathogenic *SAMD9L* missense variants [2,5,7,14] and could also be observed in our patients. In the mother, a UPD7q was found using a scatter plot of copy number and b-allele frequency, whereas a somatic missense variant was identified in the daughter that might lead to a loss of function of the respective allele. While UPD7q and loss-of-function somatic *SAMD9L* variants may restore hematopoiesis, monosomy 7 or deletion of 7q can lead to the development of myelodysplastic syndrome [14].

In this report, the patients’ clinical picture confirms that pathogenic variants in *SAMD9L* may initially present as demyelinating neuropathy and need to be considered as differential diagnosis of HMSN. Without inclusion of the daughter in trio-WES, the disease-causing variant might have been missed due to low variant allele frequency in the mother’s blood, most likely because of somatic revertant mosaicism by clonal selection. As molecular testing in patients with suspicion of a neuromuscular disorder is usually performed with DNA from peripheral blood, precautions for the detection of mosaicism of variants in *SAMD9L* in the analysis pipeline should be considered. Our report shows that the clonal disappearance of the underlying disease-causing variant may occur in the context of *SAMD9L*-associated disease without significantly abnormal blood counts.

The detection of larger CNVs and LOH (UPDs, regions of homozygosity) can be performed within the framework of a broad genetic analysis such as whole-exome/genome sequencing. To visualize the obtained CNV and LOH data, scatter plots of copy number distribution and b-allele frequency can be generated. Our report illustrates why such a scatter plot can help to improve diagnostics. Figure 2d shows an exome wide illustration with the detection of a segmental isodisomic UPD7q, including the *SAMD9L* gene, in the mother.

Identification of *SAMD9L* pathogenic variants is particularly important as there is a risk of hematological malignancies in carriers of pathogenic *SAMD9L* variants even if the magnitude of the increase in risk for myelodysplastic disorders is still unclear. Yearly follow-ups with clinical and neurological examination as well as laboratory controls (in particular complete blood count with differential) were offered to mother and daughter. Comprehensive surveys could lead to the establishment of structured clinical malignancy screening programs in the future.

*SAMD9L*-associated disease is a good example for pathogenic variants that might easily be missed and illustrates that the choice of the analyzed tissue and analysis techniques is critical to establish a correct diagnosis. In particular, the restrictions of blood as a test material must be kept in mind in unusual cases. When performing broad genetic diagnostics, tools for identifying allelic losses due to deletions or UPDs need to be implemented.

## Data Availability

The datasets generated and/or analyzed during the current study are available from the corresponding author on reasonable request.
